# Pathways for Oral and Rectal Delivery of Gold Nanoparticles (1.7 nm) and Gold Nanoclusters into the Colon: Enteric-Coated Capsules and Suppositories

**DOI:** 10.3390/molecules26165069

**Published:** 2021-08-21

**Authors:** Shabnam Hosseini, Oliver Wetzel, Kathrin Kostka, Marc Heggen, Kateryna Loza, Matthias Epple

**Affiliations:** 1 Inorganic Chemistry and Centre for Nanointegration Duisburg-Essen (CeNIDE), University of Duisburg-Essen, Universitaetsstr. 5-7, 45117 Essen, Germany; shabnam.hosseini@uni-due.de (S.H.); oliver.wetzel@uni-due.de (O.W.); kathrin.kostka@uni-due.de (K.K.); kateryna.loza@uni-due.de (K.L.); 2 Ernst Ruska Centre for Microscopy and Spectroscopy with Electrons, Forschungszentrum Jülich GmbH, 52428 Jülich, Germany; m.heggen@fz-juelich.de

**Keywords:** capsules, colon, drug delivery, gold, nanoparticles, suppositories, enteric coating

## Abstract

Two ways to deliver ultrasmall gold nanoparticles and gold-bovine serum albumin (BSA) nanoclusters to the colon were developed. First, oral administration is possible by incorporation into gelatin capsules that were coated with an enteric polymer. These permit the transfer across the stomach whose acidic environment damages many drugs. The enteric coating dissolves due to the neutral pH of the colon and releases the capsule’s cargo. Second, rectal administration is possible by incorporation into hard-fat suppositories that melt in the colon and then release the nanocarriers. The feasibility of the two concepts was demonstrated by in-vitro release studies and cell culture studies that showed the easy redispersibility after dissolution of the respective transport system. This clears a pathway for therapeutic applications of drug-loaded nanoparticles to address colon diseases, such as chronic inflammation and cancer.

## 1. Introduction

Colon-targeted drug delivery is required for the local treatment of a variety of bowel diseases, including amoebiasis, Crohn’s disease, colon cancer, and ulcerative colitis; local treatment of colon pathologies; and systemic delivery of protein and peptide drugs [1,2,3,4,5]. A colon-specific drug delivery system should be capable of protecting a drug and leading it directly into the colon. Furthermore, drug release and absorption should not occur in the stomach after oral administration [6]. In general, the colon is an appropriate absorption site for drugs and therapeutic biomolecules [7,8].

Metal nanoparticles have been the subject of many studies in drug delivery and tumor therapy because of their versatile properties [9,10,11,12,13,14,15]. Among them, gold nanoparticles are especially prominent because of their low toxicity, their good uptake by cells, and their comparatively easy synthesis and functionalization [16,17,18]. In the last decade, ultrasmall gold nanoparticles (approximately 2 nm) have gained interest due to their small size, which lies between those of molecules and proteins [11,19,20,21,22,23,24,25,26,27]. Unlike larger (plasmonic) nanoparticles, they show distinct autofluorescence, which is promising for imaging and theranostics [28,29]. They are typically prepared by reducing gold(+III) with a strong reducing agent, such as sodium borohydride, as demonstrated by Brust and Schiffrin et al. in 1994 [30,31] (see also [32], a review on these synthetic methods). It has been shown that decreasing the size of nanoparticles can improve the delivery efficiency in tumors [33,34]. Ultrasmall gold nanoparticles can also cross the blood-brain barrier [27,35].

Gold nanoclusters are even smaller (<1 nm). They have an intense autofluorescence due to molecule-like electronic transitions [36,37]. Thus, they have been proposed for biolabeling and bioimaging [38,39,40,41,42,43,44,45]. Bovine serum albumin (BSA) has been used in the synthesis of gold nanoclusters as both a reducing and a stabilizing agent [46]. It is a model protein for studying protein transport in the form of nanoclusters [47,48]. Generally, nanocarriers can transport drugs into cells for therapeutic actions, e.g., nucleic acids [49,50,51], peptides or proteins [26,52,53,54], or small molecules [55,56,57,58,59,60,61,62,63,64,65,66].

Here we report the protected delivery of freeze-dried, ultrasmall gold nanoparticles and of gold-BSA nanoclusters by stomach-acid resistant capsules and suppositories to target the colon. We also report the excellent redispersibility of the nanoparticles after release from the capsules and suppositories, including their uptake by HeLa cells, which is a prerequisite for any theranostic action.

## 2. Results and Discussion

### 2.1. Particle Synthesis and Characterization

**Figure 1:**
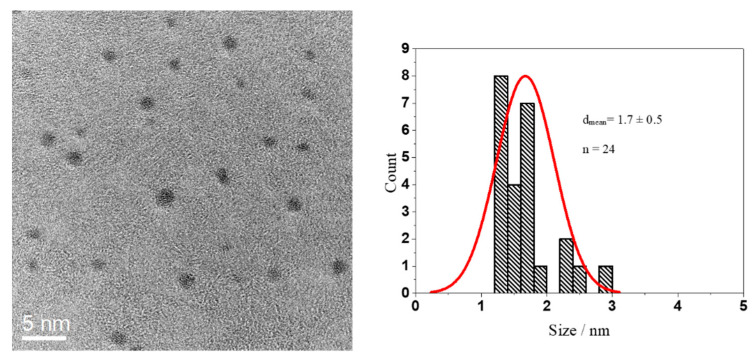
HRTEM image of Au-GSH nanoparticles (**left**) and particle size distribution (**right**).

**Figure 2:**
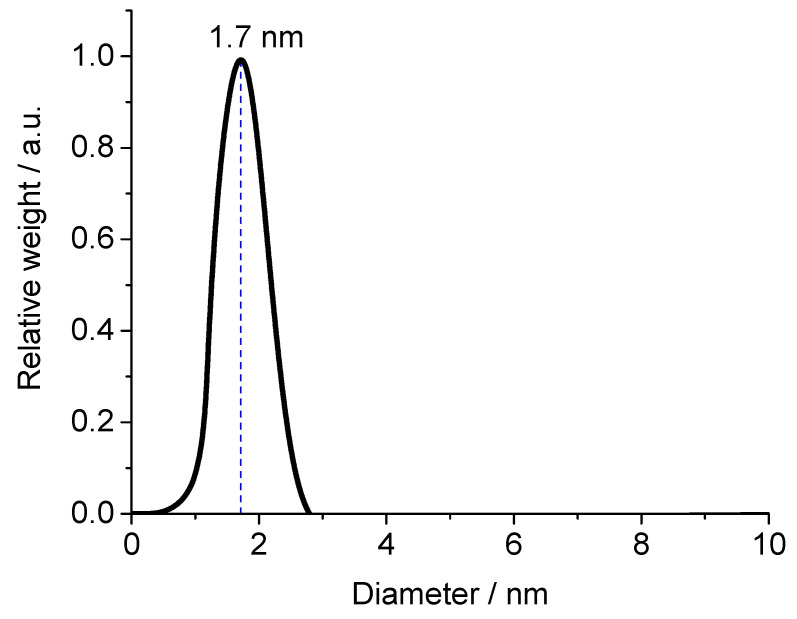
Disc centrifugal sedimentation data (DCS) of Au-GSH nanoparticles.

Glutathione-coated gold nanoparticles (Au-GSH) and BSA-functionalized gold nanoclusters (Au-BSA) were used as model compounds for the delivery of theranostic nanoparticles into the colon. Glutathione (GSH) is a tripeptide consisting of the three amino acids, glutamic acid, cysteine, and glycine. It can bind to gold via the thiol group of the central cysteine after reduction with the strong reducing agent NaBH_4_, which leads to ultrasmall gold nanoparticles [67,68,69,70,71,72]. The Au-GSH nanoparticles were characterized with respect to size and composition. High-resolution transmission electron microscopy (HRTEM) provided information about the size and morphology of the nanoparticles (Figure 1). Au-GSH particles are shown in the HRTEM image with an average core diameter of 1.7 ± 0.5 nm. This was in good agreement with the results from disc centrifugal sedimentation (DCS) that gave the hydrodynamic diameter of water-dispersed nanoparticles (Figure 2). This confirms well-dispersed nanoparticle without significant agglomeration.

**Figure 3:**
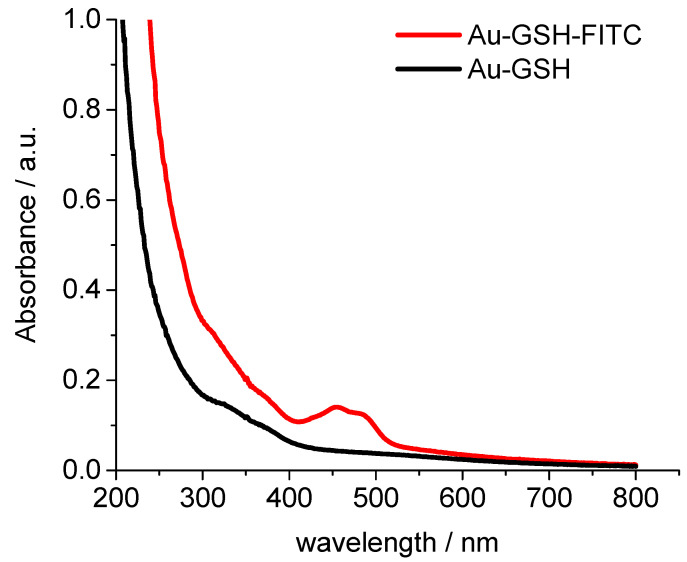
UV-Vis spectra of glutathione-functionalized ultrasmall gold nanoparticles before (black) and after labelling with NHS fluorescein (FITC) (red).

UV-Vis spectroscopy showed no surface plasmon resonance peak which would indicate the presence of bigger (plasmonic) gold particles (10 nm or more) [66]. To follow the nanoparticles inside cells, we labelled them with the fluorescent dye fluorescein isothiocyanate (FITC). The UV-Vis absorption spectrum of Au-GSH-FITC nanoparticles showed the presence of FITC with two typical absorption maxima at 450 and 490 (Figure 3).

**Figure 4:**
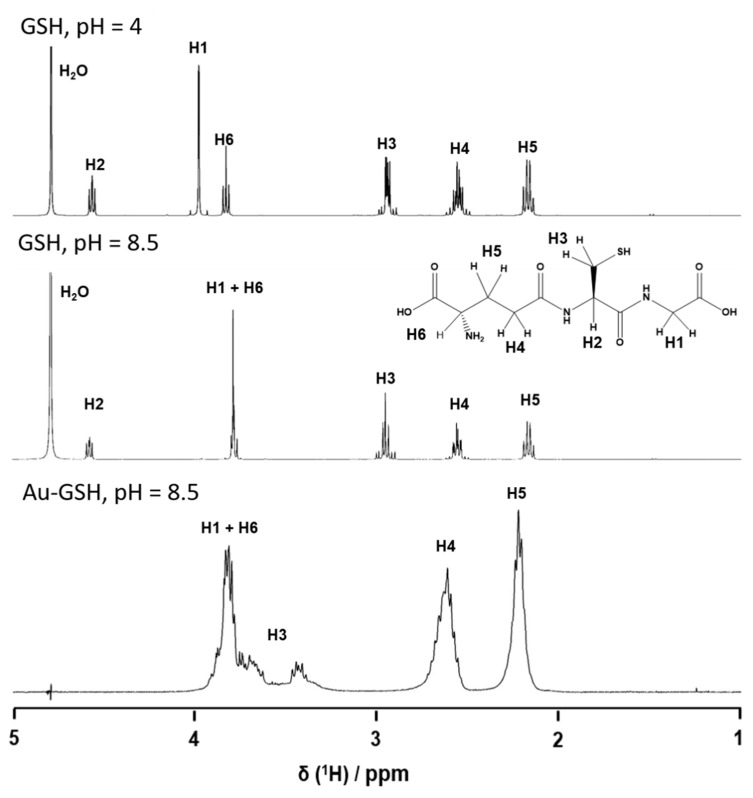
^1^H-NMR spectra of dissolved glutathione (GSH; pH 4.0 and pH 8.5) and of glutathione-functionalized ultrasmall gold nanoparticles (Au-GSH; pH 8.5).

The ligand structure is accessible by NMR spectroscopy, as the nanoparticles are ultrasmall. The ^1^H-NMR spectra of pure GSH in D_2_O at pH 4 and 8.5 showed all expected peaks and a notable pH-dependence of the chemical shifts. Figure 4 gives all relevant protons and their assignments in the spectra (see [73] for extensive NMR studies in ultrasmall Ag-GSH nanoparticles). The spectrum of Au-GSH nanoparticles shows broadened, downfield-shifted signals. This NMR peak broadening is a well-known phenomenon that is caused by the presence of a metallic nanoparticle core [73,74,75,76,77]. It depends on the distance between magnetic nucleus and the metal particle. Therefore, the signal of the (alpha) protons of the cysteine was most shifted and decreased in intensity. The NMR spectrum of the particles was measured with water suppression, which also suppressed the signal of the H2 proton due to its close proximity to the water signal. The absence of sharp NMR signals in the spectrum of the Au-GSH nanoparticles confirmed that there were no residual or detached ligands in the dispersion (Figure 4). 

**Figure 5:**
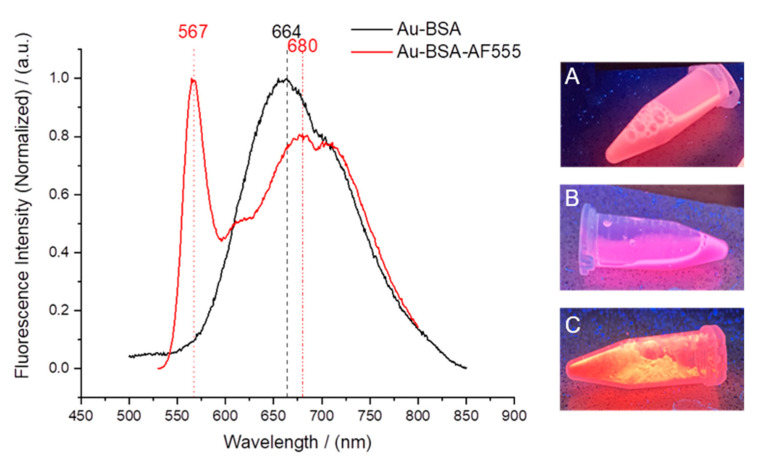
(**Left**): Fluorescence spectra of Au-BSA and Au-BSA-AF555 nanoclusters. (**Right**) Images of fluorescing nanoclusters under UV irradiation: Au-BSA dispersion (**A**), Au-BSA-AF555 dispersion (**B**), and solid Au-BSA-AF555 freeze-dried with trehalose (**C**).

Gold clusters can also be stabilized by proteins. In this respect, BSA is a model protein which also has the advantage of rendering the gold cluster autofluorescent [37,46]. To confirm the integrity of the gold-BSA cluster complex, we additionally labelled BSA with AlexaFluor555 (AF555). Thus, we could follow both the gold core and the fluorescent protein shell. Figure 5 shows the fluorescence spectra of Au-BSA and of Au-BSA-Alexa555 nanoclusters. The gold core gave rise to a broad emission peak with maxima at 664 and 680 nm. The AF555 dye led to a sharp emission maximum at 567 nm. The molar ratio of gold to BSA was determined by atomic absorption spectroscopy (AAS; Au) and by UV spectroscopy (BSA-AF555). We obtained a molar ratio Au:BSA of 4:1 after purification, in accordance with earlier studies [46,78,79,80,81]. HRTEM imaging was not possible due to the small size of the gold-BSA clusters.

### 2.2. Capsule and Suppository Loading

For colon delivery, capsules and suppositories were loaded with freeze-dried Au-GSH nanoparticles and Au-BSA nanoclusters, respectively. Table 1 and Table 2 give the full characterization data of nanoparticle-loaded capsules and suppositories. 

### 2.3. Particle Release from Capsules and Suppositories

Nanocarrier-filled coated capsules were immersed in simulated stomach medium (0.1 M HCl, pH 1) and simulated intestinal medium (MOPS buffer, 0.15 M, pH 7.1) to assess the nanoparticle release rate. The release of particles was followed by measuring the gold concentration in the immersion medium. The filled capsules were immersed in either 600 µL of simulated stomach medium or 500 µL of MOPS buffer, respectively, and aliquots of 100 µL were taken every 15 to 30 min. Then, 100 µL aqua regia was added to each aliquot to dissolve the gold nanocarriers, and the mixture was diluted with water to 3 mL and analyzed by AAS. 

**Figure 6:**
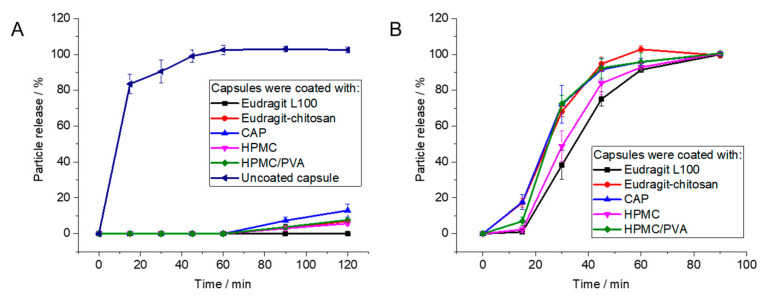
Release of Au-GSH from polymer-coated capsules by atomic absorption spectroscopy (AAS): (**A**): Coated capsules are stable under simulated stomach conditions (pH 1). (**B**): Capsules dissolve under simulated intestinal conditions (pH 7.1). Error bars indicate the standard deviation of the mean (*n* = 4).

**Figure 7:**
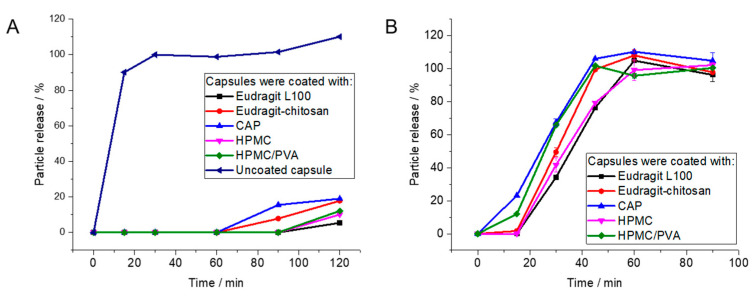
Release of Au-BSA nanoclusters from polymer-coated capsules by atomic absorption spectroscopy (AAS): (**A**): Coated capsules are stable under simulated stomach conditions (pH 1). (**B**): Capsules dissolve under simulated intestinal conditions (pH 7.1).

For uncoated gelatin capsules, rapid particle release was observed at pH 1 as the capsules were easily dissolved. The enteric-coated capsules released 12% at most after 2 h, depending on the enteric coating polymer. In contrast, the coated capsules showed rapid release of particles within 90 min at pH 7.1, i.e., under the neutral conditions in the colon (Figure 6 and Figure 7). As passage from the stomach to the colon takes hours [7,82,83,84,85], this release rate is well suited to transporting particles to the colon.

**Figure 8:**
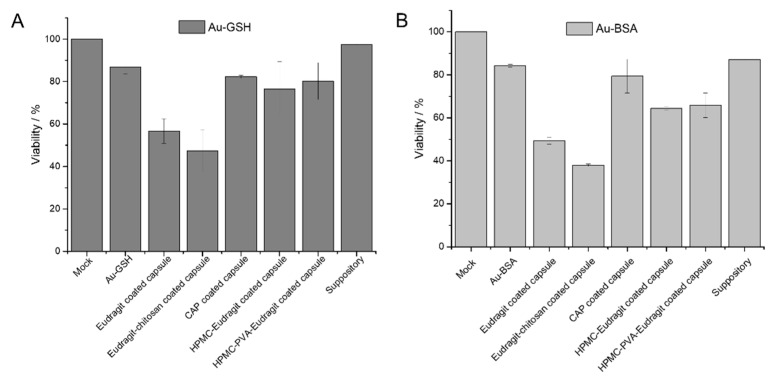
Viability of HeLa cells in the MTT assay in the presence of the components of dissolved enteric-coated capsules and suppositories, previously loaded with Au-GSH nanoparticles (**A**) and Au-BSA nanoclusters (**B**) (mean ± standard deviation; *n* = 3).

For any biomedical application, the delivery system and its cargo must be non-toxic. Therefore, the cytotoxicity of gold nanoparticles, gold nanoclusters, capsules, and suppositories was assessed with HeLa cells in MTT assays (Figure 8). For this purpose, a capsule was dissolved in 500 µL DMEM, the solution was filtered, and 250 µL of the dispersion was added to the cell culture (about 16 µg gold per well). Similarly, a half suppository was melted in 1 mL DMEM at 37 °C, and 250 µL of the dispersion was given to the cell culture (15.5 µg gold per well). As control, 35 µg of water-dispersed gold nanocarriers was given to the cell culture. Except for Eudragit and Eudragit-chitosan in combination with the gelatin capsules, all components were highly cytocompatible. The cytotoxicity appears to be a synergetic effect of Eudragit L100 and gelatin, as already observed [85].

### 2.4. Uptake of Particle Released from Capsules and Suppositories by Cells

**Figure 9:**
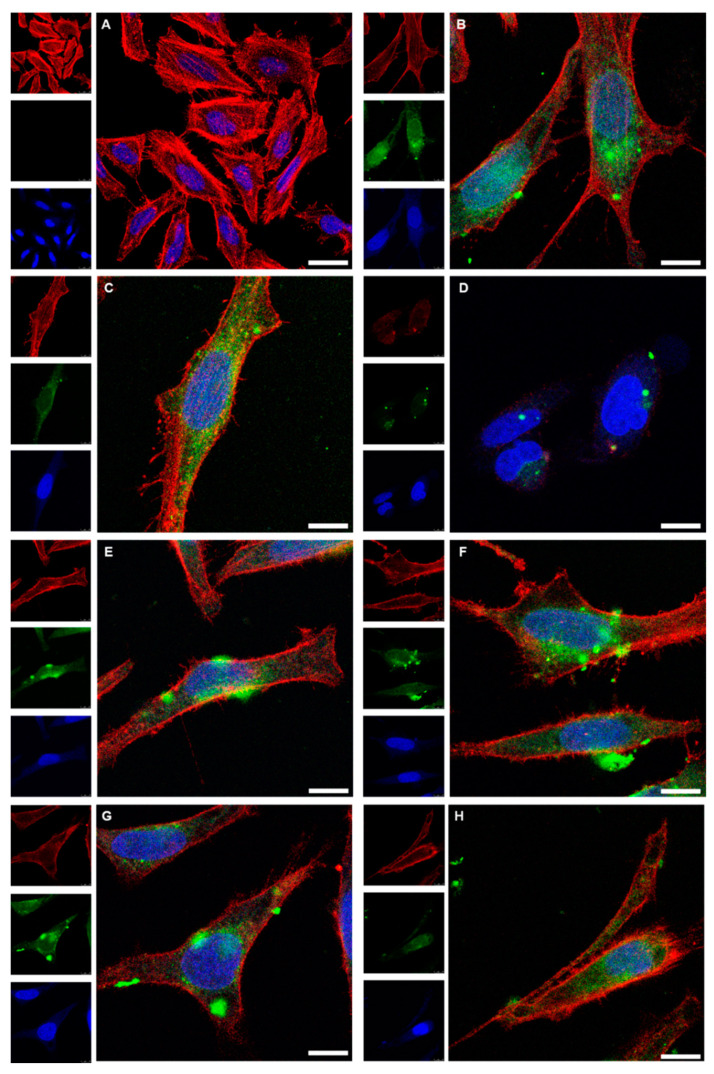
Confocal laser scanning microscopy images of HeLa cells after 24 h cultivation (control; (**A**)), and after incubation for 24 h with dispersed Au-GSH-FITC nanoparticles (70 µg mL^−1^) (**B**). The uptake of Au-GSH-FITC nanoparticles from coated gelatin capsules after dissolution is shown in (**C**) for Eudragit (88 µg mL^−1^), (**D**) for Eudragit-chitosan (65 µg mL^−1^), (**E**) for cellulose acetate phthalate (CAP; 60 µg mL^−1^), (**F**) for hydroxypropylmethylcellulose-Eudragit (HPMC-Eudragit; 60 µg mL^−1^), and (**G**) for hydroxypropylmethylcellulose-polyvinylalcohol-Eudragit (HPMC-PVA-Eudragit; 72 µg mL^−1^). (**H**) The uptake of Au-GSH-FITC nanoparticles after release from a hard-fat suppository (62 µg mL^−1^). Red: actin (cytoskeleton); blue: cell nucleus; green: Au-GSH-FITC nanoparticles. Scale bar 10 µm. All given concentrations refer to gold in the cell culture well.

The nanoparticles and nanoclusters were well redispersed after dissolution of the capsule or the suppository and easily taken up by cells, underscoring their ability to act as drug delivery vehicles. Figure 9 shows cell uptake experiments of as-prepared and of released Au-GSH nanoparticles with HeLa cells. Confocal laser scanning microscopy showed a strong green fluorescence of FITC in the cytosol. The nanoparticles were released from the enteric-coated capsules and suppositories and well taken up by HeLa cells, except for Eudragit-chitosan-coated capsules. In that case, a possible interaction among the two polymers, the dissolved gelatin capsule, and the particles may have led to agglomeration (the cells were washed before imaging, removing most extracellular particles). Similar results were also obtained for capsules and suppositories containing drug-loaded calcium phosphate nanoparticles that were also successfully taken up by the HeLa and Caco-2 cells after dissolution of capsules [85] or suppositories [86]. This indicated that the colloidal dispersibility of the nanoparticles was not constrained by freeze-drying and capsule/suppository dissolution, so that they could still easily penetrate the cell membrane.

**Figure 10:**
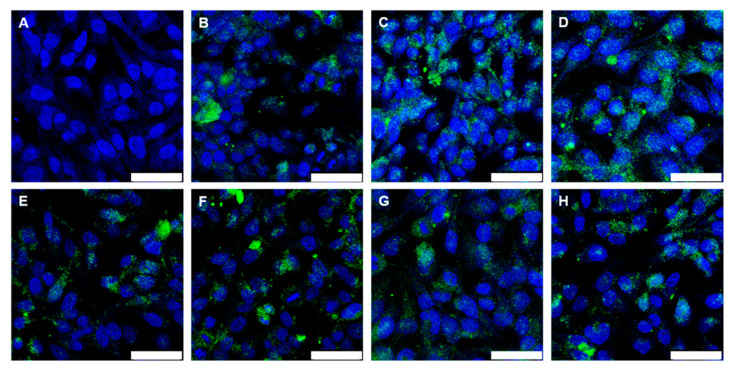
Confocal laser scanning microscopy images of HeLa cells after 24 h cultivation (control; (**A**)) and after 24 h incubation with Au-BSA nanoclusters (43 µg mL^−1^ Au) (**B**). The uptake of Au-BSA nanoclusters from dissolved capsules is shown in (**C**) for Eudragit (42 µg mL^−1^ Au), (**D**) for Eudragit-chitosan (42 µg mL^−1^ Au), (**E**) for CAP (45 µg mL^−1^ Au), (**F**) for HPMC-Eudragit (50 µg mL^−1^ Au), and (**G**) for HPMC-PVA-Eudragit (47 µg mL^−1^ Au). (**H**) The uptake of Au-BSA nanoclusters after release from a suppository (52 µg mL^−1^ Au). Blue: nucleus; green: Au-BSA nanocluster autofluorescence. Scale bar 50 µm. All given concentrations refer to gold in the cell culture well.

The results were comparable for Au-BSA-AF555 nanoclusters (Figure 10). Confocal laser scanning microscopy showed the green autofluorescence of gold nanoclusters that easily penetrated the cell membranes due to their small size. The nanoclusters were released from the enteric coated capsules and suppositories, obviously in a well-dispersed form, and taken up by the HeLa cells. Again, the formulation of the nanoclusters by the sequence of freeze-drying and incorporation into capsules/suppositories, followed by the dissolution of the carrier system under colon-specific conditions, did not compromise their high degree of autofluorescence and their small particle size in the dispersion, leading to good uptake by cells.

**Figure 11:**
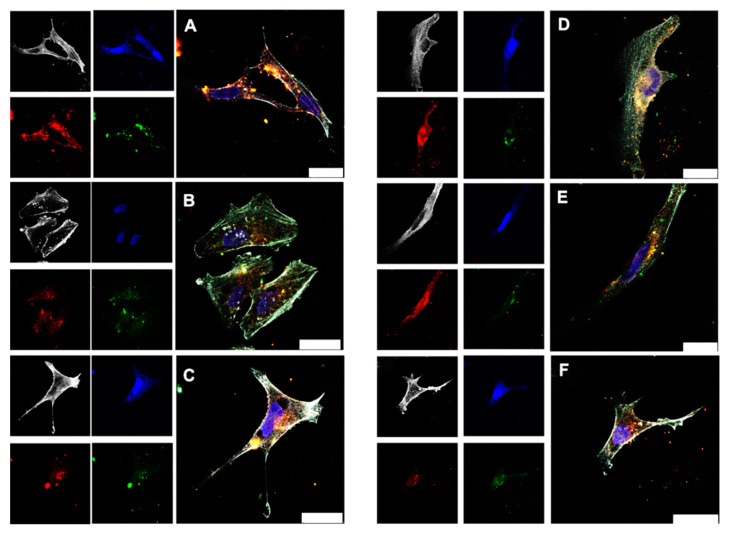
Confocal laser scanning microscopy images of HeLa cells incubated for 24 h with dispersed Au-BSA-AF555 nanoclusters (13.3 µg mL^−1^ Au) (**A**). The uptake of Au-BSA-AF555 nanoclusters from dissolved capsules is shown in (**B**) for Eudragit (14.5 µg mL^−1^ Au), in (**C**) for CAP (15.4 µg mL^−1^ Au), in (**D**) for HPMC-Eudragit (17.0 µg mL^−1^ Au), and in (**E**) for HPMC-PVA-Eudragit (16.5 µg mL^−1^ Au). (**F**) The uptake of Au-BSA-AF555 nanoclusters after release from a suppository (22 µg mL^−1^ Au). Grey: actin; blue: nucleus; green: Au-BSA autofluorescence; red: Au-BSA-AF555 fluorescence from AF555. Scale bar 25 µm.

Protein-coated gold nanoclusters can be used to transport proteins into cells [87]. To monitor the transport of the protein BSA with the ultrasmall gold nanoclusters in more detail, cell uptake studies were performed with HeLa cells and Au-BSA-AF555 nanoclusters (Figure 11). Confocal microscopy showed that the gold autofluorescence and the BSA-AF555 fluorescence were mostly co-localized; i.e., gold nanoclusters and protein were taken up together. This demonstrates the good dispersibility of the gold nanoclusters together with the protein BSA after all formulation steps.

To overcome the acidic medium of stomach for colon drug delivery, combinations of different pH-dependent polymers have been used. In the following, we discuss some of the most recent developments. Crowe et al. prepared tablets with a coating of Eudragit L100 for the colonic delivery of the drug V565 (an antibody against TNF-α). This tablet showed sustained drug release at pH 6, yet no drug release during incubation with an acidic medium for 2 h [88]. Nguyen et al. used the biopolymer zein (a protein derived from corn) in combination with Kollicoat^®^ MAE 100P (a methacrylic acid copolymer) to coat capsules. This combination resulted in drug release in the upper gastrointestinal (GI) tract [89]. Berardi et al. used zein to achieve sustained release of certain drugs. After filling hard gelatin capsules with a physical mixture of zein and the drug propranolol in different ratios, zein formed a polymer matrix that retarded the drug release [90]. Barbosa et al. prepared different enteric capsule coatings to target different regions of the GI tract. They used cellulose derivatives (HPMC ASLF and HP-55) along with acrylic/methacrylic acid derivatives (Eudragit^®^ L100 and Eudragit^®^ S100) [91]. Chauhan et al. reported protein nanoparticle (PNP)-loaded suppositories for colon targeting with the drug metronidazole (MZ), to achieve a sustained release effect. In vitro drug release testing showed that protein nanoparticle-loaded suppositories showed better release then MZ-loaded suppositories [92]. Varum et al. developed the OPTICORE™ coating technology as a novel enteric coating system. Here, a combination of pH-triggered and enzymatic degradation was used. They showed that this system offers significant advantages for accurate drug delivery in the colons of ulcerative colitis patients [93]. Oshi et al. coated dexamethasone microcrystals with several layers with a layer-by-layer coating technique. Chitosan, oligosaccharide, alginate, and Eudragit S 100 (ES) as a pH-resistant polymer were added in order to achieve colon-targeted delivery of dexamethasone as a model drug [94]. Bazan et al. developed celecoxib-loaded Eudragit microparticles and carried out in vivo studies in a rat model. They found controlled release of celocoxib (a COX-2 inhibitor, a small molecule drug), and significant decreases in colon inflammation and inflammation markers [95]. 

In general, the combination of different enteric coating systems can lead to an adjustable time for specific drug release [85]. Thereby, specific areas of the colon can be targeted, resulting in more precise treatments. Here we report for the first time how ultrasmall gold nanoparticles and nanoclusters can be incorporated into pH-sensitive enteric coated capsules and suppositories to achieve site-specific drug delivery. After carrier degradation, they are well dispersed for cellular uptake and delivery of drug cargo. The incorporation of ultrasmall gold nanoparticles—which were also reported to enter the cell nucleus in some cases [96]—into gelatin capsules and suppositories, opens up new opportunities in drug delivery: using nanoparticles and nanoclusters as drug and protein carriers.

## 3. Materials and Methods

### 3.1. Materials

Size “M” gelatin capsules for oral delivery into mice were obtained from Torpac Inc. (Fairfield, CT, USA; volume 4 µL; external diameter 1.27 mm; maximum length after closing 8.4 mm; capsule surface 31 mm^2^; capsule weight about 2 mg; disintegration time in the stomach about 10 min; all data according to Torpac). In order to cover the capsules with enteric polymers, Eudragit^®^ L100 (denoted as Eudragit in the following) was obtained from Evonik Industries (Darmstadt, Germany) in pharmaceutical grade and used without further purification. (Hydroxypropyl)methylcellulose (viscosity 80–120 cP; *M* = 26 kDa; denoted as HPMC in the following), cellulose acetate phthalate (*M* = 2.5 kDa; denoted as CAP in the following), poly(vinylalkohol) (87–90% hydrolysed, *M* = 30–70 kDa; denoted as PVA in the following), chitosan (2-amino-2-deoxy-(1,4)-β-D-glucopyranan, poly-(1,4-β-D-glucopyranosamine) (viscosity > 400 mPa s), and D-(+)-trehalose dihydrate (>99%) were obtained from Sigma-Aldrich (Taufkirchen, Germany) and used without further purification. Adeps solidus (pharmaceutical hard fat, Witepsol^®^W25) was obtained from Caesar and Loretz GmbH, Mainz, Germany). Tetrachloroauric(III)acid (HAuCl_4_) was prepared by dissolution of gold in aqua regia. Sodium borohydride (NaBH_4_, Fluka, purity > 96%, Schwerte, Germany), L-glutathione (purity > 98%, Sigma-Aldrich, Darmstadt, Germany), Pierce NHS-fluorescein (5/6-carboxyfluorescein succinimidyl ester, >90% by HPLC; Thermo Scientific, Rockford, IL, USA), bovine serum albumin (BSA, Serva Electrophoresis GmbH, Heidelberg, Germany), and sodium hydroxide (>98.6%; VWR Prolabo, Hannover, Germany) were used in the nanoparticle syntheses.

### 3.2. Instruments

Scanning electron microscopy was performed with an ESEM Quanta 400 instrument (FEI, Munich, Germany) with gold/palladium-sputtered samples. Confocal laser scanning microscopy was performed with an SP8 Falcon instrument (Leica, Wetzlar, Germany) with a 63× water objective. The laser wavelength was 405 nm for Hoechst33342 excitation (emission: 440–480 nm), 488 nm for FITC excitation (emission: 500–520 nm), and 640 nm for Alexa-660 excitation (emission: 657–720 nm). Centrifugation was performed with a Heraeus Fresco 21 instrument (Thermo Scientific, Langenselbold, Germany). Ultracentrifugation was performed with a Sorvall WX Ultra Series centrifuge (Thermo Electron Corporation, Schwerte, Germany). Uptake efficiencies were determined by transmission light microscopy and fluorescence microscopy with a BZ900 microscope (Keyence, Neu Isenburg, Germany). The viability of the cells was analyzed with an MTT test by spectrophotometric analysis with a Multiscan FC instrument (Thermo-Fisher scientific, Vantaa, Finland) at *λ* = 570 nm. Freeze-drying was performed with an Alpha 2–4 LSC instrument (Christ, Osterode am Harz, Germany). Gold was determined by atomic absorption spectroscopy (AAS) with a Thermo Electron (Karlsruhe, Germany) M-Series spectrometer (graphite tube furnace according to DIN EN ISO/IEC 17025:2005) after dissolving the particles in aqua regia. Differential centrifugal sedimentation (DCS) was performed with a CPS Instruments DC 24,000 disc centrifuge (24,000 rpm). A density gradient was prepared with two sucrose solutions (8 and 24 wt.%) and capped with 0.5 mL dodecane. Poly(vinylchloride) latex in water with a particle size of 483 nm (CPS Instruments, Oosterhout, The Netherlands) was used as a calibration standard. A volume of 100 µL was used for both calibration and sample measurement. High-resolution transmission electron microscopy (HRTEM) was performed with an aberration-corrected FEI Titan transmission electron microscope equipped with a Cs-probe corrector (CEOS, Heidelberg, Germany), operated at 300 kV. ^1^H-NMR spectra were recorded with an Avance III 600 MHz spectrometer (Bruker, Rheinstetten, Germany) equipped with a Prodigy cryoprobe head. The spectra were recorded with simultaneous suppression of the water signal by excitation sculpting due to the low ligand concentration in the aqueous dispersions. The excited spectral range had a width of approximately 0.6 ppm. Fluorescence spectra were measured with a Cary Eclipse spectrophotometer (Agilent, Waldbronn, Germany) in a quartz cuvette.

### 3.3. Synthesis

All glassware was boiled beforehand with aqua regia and thoroughly rinsed with ultrapure water. All syntheses were carried out with ultrapure water (Purelab ultra instrument, ELGA) and at ambient temperature unless otherwise noted. 

For the synthesis of glutathione-protected ultrasmall gold nanoparticles (denoted as Au-GSH in the following) by an adapted Brust-Schiffrin synthesis [31,32], 2.5 mL tetrachloroauric(III) acid (20 mM, 50 µmol) and 46 mg glutathione (150 µmol) were dissolved in 30 mL degassed water. After 30 min stirring time, 2 mL of a freshly prepared solution of sodium borohydride (250 mM, 500 µmol) was added. The mixture was stirred for another 30 min. The product was purified by spin filtration (3 kDa molecular weight cutoff; Amicon; Merck, Darmstadt, Germany) for 40 min at 4000 rpm (2500 *g*). The gold nanoparticles were precipitated at low pH (ca. 3). The precipitated product was redispersed in 15 mL 0.1 mM NaOH and spin-filtered/washed twice. NHS-fluorescein (5/6-carboxyfluorescein succinimidyl ester) was used for reactive amino-labelling of Au-GSH nanoparticles. Then, 10 mg of NHS-fluorescein was dissolved in 100 µL DMF and added to a solution of 1 mg freeze dried particles dissolved in 5 mL borate buffer (pH = 8.5). The mixture was reacted for 48 h at 8 °C, spin-filtered, redispersed, and washed several times for 40 min at 4000 rpm (2500 *g*) to remove the nonreacted NHS-fluorescein until the filtrate was colorless. These particles are denoted as Au-GSH-FITC in the following.

For the synthesis of fluorescent gold-BSA nanoclusters (denoted as Au-BSA in the following) according to Xie et al. [46], an aqueous HAuCl_4_ solution (5 mL, 10 mM, 37 °C) was added to a BSA solution (5 mL, 50 mg mL^−1^, 37 °C) under vigorous stirring. A NaOH solution (0.5 mL, 2 M) was added after 2 min, and the mixture was reacted under stirring at 37 °C for 16 h. BSA acts both as reducing and stabilizing agent. The basic pH was necessary to avoid the formation of larger gold nanoparticles, as demonstrated by Xie et al. [46]. The nanoclusters were isolated by ultracentrifugation at 66,000 *g* for 12 h. The Au-BSA nanoclusters were concentrated in the lower part of the centrifugal tube. The supernatant was decanted to isolate the nanoclusters. BSA was fluorescently labelled with AlexaFluor™ 555 (AF555, C2 Maleimide). In addition, 200 µL AF555-C2-maleimide was added to a BSA solution (10 mL, 50 mg mL^−1^, and 37 °C), stirred for 5 min, and kept for 1 h at room temperature to complete the reaction. The labelled BSA-AF555 was centrifuged three times at 14,000 *g* for 15 min with 500 µL spin filters and then used for the synthesis of Au-BSA-AF555 nanoclusters as described above. 

### 3.4. Freeze-Drying

Trehalose was added as cryoprotectant to the water-dispersed particles in the weight ratio of 100 to 1 (trehalose:nanoparticles). The solution was lyophilized. Afterwards the dried mixture was analyzed with UV-Vis spectroscopy to calculate the amount of FITC or BSA-AF555 in the whole sample, using a previously recorded calibration curve. The amount of gold was analyzed by dissolving a defined quantity of the dried product in aqua regia and water, followed by AAS analysis.

### 3.5. Preparation of Suppositories

Cylindrical suppositories (2 × 16 mm^2^) were prepared from hard fat with dispersed freeze-dried nanoparticles in a PTFE mould as described earlier [86]. Briefly, hard fat was first melted in a syringe at 37 °C, mixed with the freeze-dried nanoparticles (about 0.2 g of freeze-dried particles/trehalose 1:100 per 1 g of hard fat), injected into the PTFE mold, and then solidified at 4 °C.

### 3.6. Preparation of Enteric-Coated Capsules

Capsules were prepared, filled, and coated as described in [85]. Briefly, the capsules were filled with a mixture of freeze-dried nanoparticles or nanoclusters with the cryoprotectant trehalose and then coated with either Eudragit, Eudragit/chitosan, cellulose acetate phthalate (CAP), hydroxypropylmethylcellulose (HPMC)/Eudragit, or HPMC/polyvinyl alcohol (PVA)-Eudragit. The loading with nanoparticles/trehalose and the amount of the coating polymers were determined gravimetrically. Release experiments at low and at neutral pH were carried out as reported earlier [85]. 

### 3.7. Cell Culture Studies

Cell culture studies with Au-GSH-FITC nanoparticles and Au-BSA-Alexa555 nanoclusters were carried out with the human cervix carcinoma cell line HeLa [97]. The cells were cultured in Dulbecco’s modified eagle’s medium (DMEM), supplemented with 10% fetal bovine serum (FBS, Gibco), 100 U mL^−1^ penicillin, and 100 U mL^−1^ streptomycin at 37 °C in a humidified atmosphere with 5% CO_2_. The nanoparticle-loaded carriers were dissolved in 0.5 mL cell culture medium for 15 min (for suppositories) to 2 h (for capsules) in a water bath in 37 °C before the addition to the cells. The cytotoxicity was determined with a 3-(4,5-dimethylthiazol-2-yl)-2, 5-diphenyltetrazolium bromide (MTT) cytotoxicity assay. The cells were trypsinized and seeded in a 24-well culture dish with 2.5 × 10^4^ cells per well in 500 µL cell culture medium 24 h prior to the experiments. Afterwards, the cells were washed three times with 500 µL PBS and incubated with 300 µL MTT solution (1 g L^−1^) for 1 h at 37 °C. Then, the MTT solution was replaced by 300 µL DMSO and incubated for 30 min. Finally, sample triplicates of the DMSO solution were transferred to a 96-well plate (100 µL aliquots) for spectrophotometric analysis at 570 nm. The relative cell viability was calculated by comparison to a control group of untreated cells.

For uptake studies, the cells were trypsinized and seeded in a glass bottom dish with 10^4^ cells per well in 250 µL of cell culture medium, 24 h prior to the experiments. The cells were incubated for 24 h with the nanoparticles. The cells were then washed three times with 300 µL PBS. After the completed incubation, the cells were fixed with 100 µL 4% aqueous formaldehyde for 20 min at room temperature and washed again three times with 300 µL PBS each. Prior to actin staining, the cells were permeabilized with 150 µL 0.1% Triton X-100 for 5 min and washed twice with 300 µL PBS each. For actin staining, the cells were incubated with 150 µL of a solution of 25 µg mL^−1^ AlexaFluor^®^ 660-phalloidin (Invitrogen, Karlsruhe, Germany) in PBS with 1% bovine serum albumin, and then washed three times with 150 µL PBS (each wash). The cell nucleus was stained with 150 µL of a 10 µg mL^−1^ solution of Hoechst33342 (life technologies, Eugene, OR, USA) for 15 min. The cells were washed three times with 300 µL PBS each, stored in 250 µL PBS, and finally analysed by confocal laser scanning microscopy.

## 4. Conclusions

We have presented strategies for the oral and rectal transport of ultrasmall gold nanoparticles and of gold nanoclusters into the colon. In order to deliver theranostic nanoparticles and nanoclusters by oral intake and to protect them from gastric acid, they can be freeze-dried and put into a gelatin capsule that is coated with an enteric coating polymer. The enteric coated capsules are stable under the stomach’s acidic conditions (pH 1). Another possibility is the incorporation of nanocarriers into hard-fat suppositories. The nanoparticles and nanoclusters are well redispersed in cell culture medium (DMEM) after dissolution of capsule or suppository and well taken up by HeLa cells. Although Eudragit L100-chitosan as one of our enteric coating options was efficient for protection against acids, uptake of the nanoparticles into cells did not occur, and it was significantly cytotoxic. In contrast, capsules coated with Eudragit L100, cellulose acetate phthalate, hydroxypropylmethyl cellulose-Eudragit, or hydroxypropylmethyl/cellulose-polyvinylalcohol-Eudragit are well suited for efficient particle uptake by cells. Thus, enteric coated capsules and hard-fat suppositories are efficient carriers of drug-loaded gold nanoparticles and gold-protein nanoclusters for potential biomedical applications involving local or systemic drug delivery, including delivery of proteins. 

## Figures and Tables

**Table 1:** Characterization of gelatin capsules, loaded with either Au-GSH nanoparticles or Au-BSA nanoclusters. Given are weight percentages with respect to the total weights of nanocarriers, including trehalose (±indicates the standard deviation).

Empty capsule weight/mg	2.2 ± 0.5
Capsule content of Au-GSH-FITC + trehalose/mg	3.3 ± 0.5 (100%)
Capsule content of Au/µg	34 ± 5 (1.05%)
Capsule content of FITC/µg	4.0 ± 0.6 (0.12%)
Capsule content of Au-BSA-AF555 + trehalose/mg	3.3 ± 0.5 (100%)
Capsule content of Au/µg	6.3 ± 0.9 (0.19%)
Capsule content of AF555/µg	0.2 ± 0.05 (0.007%)
Capsule content of BSA/mg	0.5 ± 0.1 (14.6%)

**Table 2:** Characterization of hard-fat suppositories, loaded with either Au-GSH nanoparticles or Au-BSA nanoclusters. Given are weight percentages with respect to the total weight of the suppository (±indicates the standard deviation).

Total suppository weight/mg	60 ± 2 (100%)
Suppository content of hard fat/mg	48 ± 2 (80.3%)
Suppository content of Au-GSH-FITC + trehalose/mg	11.8 ± 0.5 (19.7%)
Suppository content of Au/µg	124 ± 5 (0.2%)
Suppository content of FITC/µg	14.2 ± 0.6 (0.02%)
Suppository content of Au-BSA-AF555 + trehalose/mg	11.8 ± 0.5 (19.7%)
Suppository content of Au/µg	22.5 ± 1.0 (0.04%)
Suppository content of AF555/µg	0.9 ± 0.05 (0.002%)
Suppository content of BSA/mg	1.7 ± 0.1 (2.8%)

## Data Availability

Primary data are available upon request from the corresponding author.

## Sample Availability

Samples of the compounds are not available from authors.
